# Activation of skeletal muscle–resident glial cells upon nerve injury

**DOI:** 10.1172/jci.insight.143469

**Published:** 2021-04-08

**Authors:** Daisy Proietti, Lorenzo Giordani, Marco De Bardi, Chiara D’Ercole, Biliana Lozanoska-Ochser, Susanna Amadio, Cinzia Volonté, Sara Marinelli, Antoine Muchir, Marina Bouché, Giovanna Borsellino, Alessandra Sacco, Pier Lorenzo Puri, Luca Madaro

**Affiliations:** 1 IRCCS Fondazione Santa Lucia, Rome, Italy.; 2 Department of Anatomy, Histology, Forensic Medicine and Orthopedics, University of Rome “la Sapienza”, Rome, Italy.; 3 Sorbonne Université, INSERM UMRS 974, Association Institut de Myologie, Centre de Recherche en Myologie, Paris, France.; 4 CNR, National Research Council, Institute for Systems Analysis and Computer Science, Rome, Italy.; 5 CNR, National Research Council, Institute of Biochemistry and Cell Biology, Monterotondo Scalo, Rome, Italy.; 6 Development, Aging and Regeneration Program, Sanford Burnham Prebys Medical Discovery Institute, La Jolla, California, USA.

**Keywords:** Muscle Biology, Neuromuscular disease, Skeletal muscle

## Abstract

Here, we report on the identification of Itga7-expressing muscle-resident glial cells activated by loss of neuromuscular junction (NMJ) integrity. Gene expression analysis at the bulk and single-cell level revealed that these cells are distinct from Itga7-expressing muscle satellite cells. We show that a selective activation and expansion of Itga7^+^ glial cells occur in response to muscle nerve lesion. Upon activation, muscle glial–derived progenies expressed neurotrophic genes, including nerve growth factor receptor, which enables their isolation by FACS. We show that activated muscle glial cells also expressed genes potentially implicated in extracellular matrix remodeling at NMJs. We found that tenascin C, which was highly expressed by muscle glial cells, activated upon nerve injury and preferentially localized to NMJ. Interestingly, we observed that the activation of muscle glial cells by acute nerve injury was reversible upon NMJ repair. By contrast, in a mouse model of ALS, in which NMJ degeneration is progressive, muscle glial cells steadily increased over the course of the disease. However, they exhibited an impaired neurotrophic activity, suggesting that pathogenic activation of glial cells may be implicated in ALS progression.

## Graphical abstract

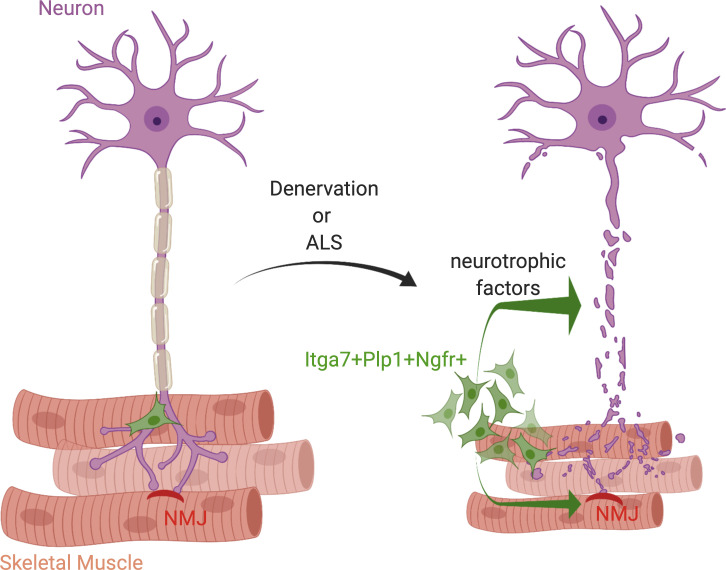

## Introduction

Skeletal muscle homeostasis is maintained by a large network of muscle-resident cells that coordinate the response to homeostatic perturbations, such as muscle or nerve injury (1, 2). Among these cells, muscle satellite cells (MuSCs) are the bona fide muscle stem cells that exist in a quiescent state during homeostasis in adult tissues and become activated in response to acute muscle damage or in chronic degenerative conditions (3–8). In addition to their absolute requirement for regeneration of injured muscle, recent studies have reported that MuSCs may also participate in the maintenance of neuromuscular junction (NMJ) integrity and regeneration upon nerve injury (9, 10). Nerve injury and repair are events closely associated with the process of muscle regeneration; however, the precise contribution of MuSCs to NMJ regeneration is poorly understood. Likewise, whether additional muscle-resident cell types contribute to neurite growth and extension toward regenerating fibers to restore functional NMJs remains unknown.

We and others have recently reported on the activation of muscle-resident mesenchymal progenitors, the fibroadipogenic progenitors (FAPs), and their expansion following denervation (11). In addition to FAPs, resident macrophages have also been implicated in the process of NMJ repair following denervation or injury (9, 10, 12, 13). Interestingly, alterations in function and number of MuSCs and FAPs have been observed in neuromuscular disorders such as ALS, in which loss of NMJ integrity occurs progressively (14, 15).

In postnatal life, the disruption of skeletal muscle–nerve crosstalk leads to muscle atrophy and fibrosis (16–18), eventually leading to irreversible paralysis in conditions of complete nerve loss (i.e., traumatic spinal cord injury) or progressive loss of NMJ (e.g., chronic neuromuscular disorders) (19). Therefore, identification of the cell types activated by nerve injury and an improved understanding of their functional interactions is imperative in order to develop novel therapeutic strategies to counter the effect of muscle denervation.

In this study, we performed gene expression analysis in both bulk and single cells isolated from limb muscle following nerve injury to identify the cellular players that might contribute to nerve repair.

## Results

### Nerve injury activates a neurotrophic program in Itga7^+^ nonsatellite cells.

**Figure 1:**
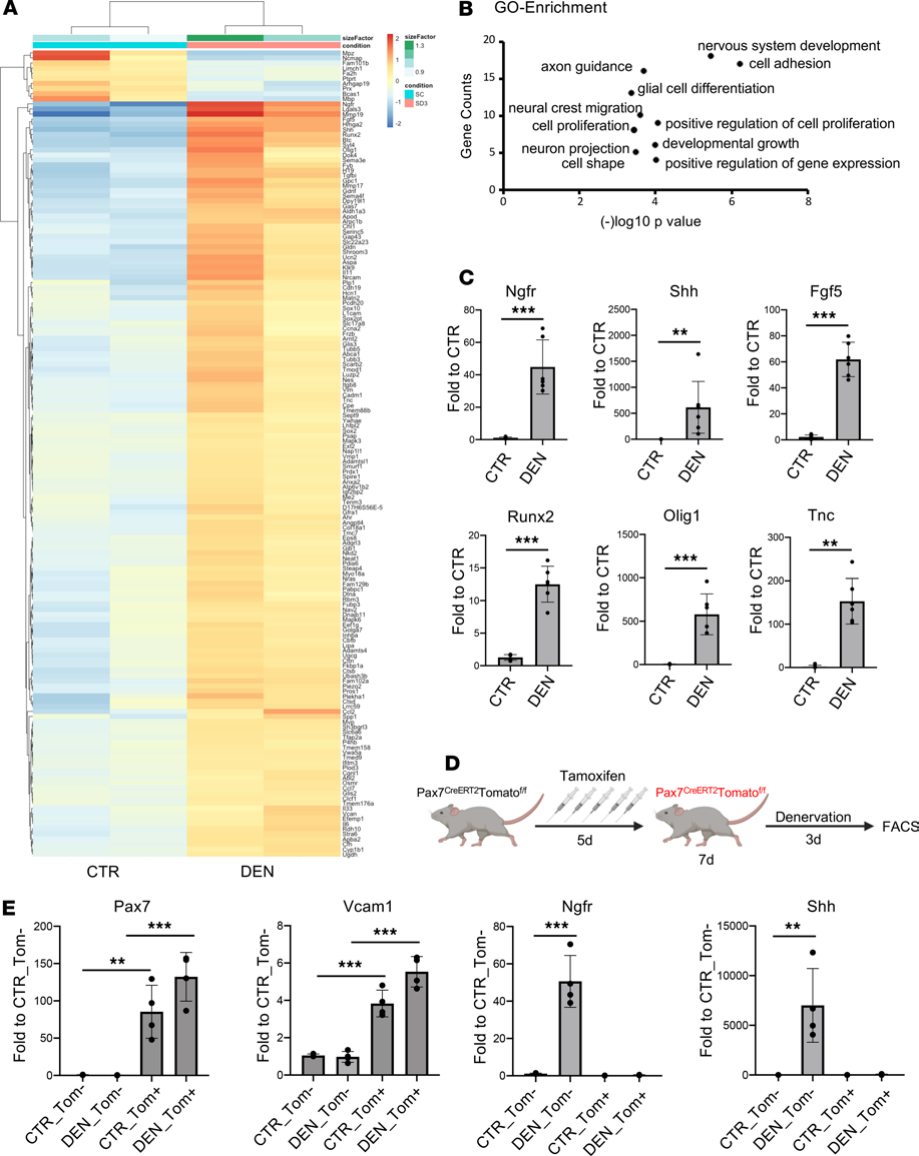
Activation of a neurotrophic signaling pathway in the Itga7^+^Sca1^–^Ln^–^ myogenic cell fraction. (**A**) Heatmap representation of genes significantly deregulated (*P* adj < 0.001) in ltga7^+^Sca1^–^Ln^–^ freshly isolated cells derived from denervated (cut) muscle at 3 days after nerve lesion (*n* = 2). (**B**) Gene ontology (GO) enrichment in biological function of genes significantly deregulated (*P* adj < 0.001) in ltga7^+^Sca1^–^Ln^–^ cells derived from 3 days denervated muscle. (**C**) qPCR analysis for Ngfr, Shh, Fgf5, Runx2, Olig1, and Tnc expression in freshly isolated ltga7^+^Sca1^–^Ln^–^ cells derived from control and 3 days reversible denervated (crush) muscle. GAPDH was used as housekeeping gene (*n* ≥ 5, values represent mean ± SD, **P < 0.01, ***P < 0.001; by Student’s *t* test (Ngfr, Fgf5, Olig1, Runx2, and Shh) or by Mann-Whitney test (Tnc). (**D**) Working model of tamoxifen-induced (Tmx-induced) in vivo treatment. (**E**) qPCR analysis for Pax7, Vcam1, Ngfr, and Shh expression in freshly isolated Tomato^+^ and Tomato^–^ cells derived from control and 3 days denervated muscle of Tmx-treated PAX7.Cre_tdTomato mice. GAPDH was used as housekeeping gene (*n* = 4, values represent mean ± SD, **P < 0.01, ***P < 0.001; by 1-way ANOVA Tukey’s multiple-comparison test).

The contribution of Itga7-expressing MuSCs to the maintenance of NMJ integrity and regeneration upon nerve injury was previously reported by Liu et al. (9, 10). These studies prompted our interest in the transcriptional profiles of Itga7^+^ cells isolated from limb muscles of mice either unperturbed or exposed to nerve injury. To this end, we performed RNA-Seq analysis on Itga7^+^Sca1^–^Ln^–^ cells (herein referred to as Itga7^+^ cells) isolated by FACS from limb muscles of 3-month-old mice, at 3 days after denervation by sciatic nerve severing, and compared with Itga7^+^ cells isolated from control mice. Heatmap comparison revealed extensive alterations of the transcriptional profile in Itga7^+^ cells isolated after nerve injury, compared with unperturbed controls, with a clear bias toward upregulated genes, which accounted for the vast majority of the differentially expressed genes (Figure 1A). Notably, most of the upregulated genes were those associated with neuronal growth and repair pathways, including nerve growth factor receptor (Ngfr), sonic hedgehog (Shh), tenascin C (Tnc), neuronal cell adhesion molecule (Nrcam), glial cell–derived neurotrophic factor (Gdnf), and the glial lineage–specific transcription factors oligodendrocyte lineage genes (Olig1) (Figure 1A). Other genes of alternative mesenchymal lineages, but recently implicated in neurogenesis (e.g., Runx2) (20), were also found upregulated in Itga7^+^ cells isolated following nerve injury. Moreover, gene ontology analyses of RNA-Seq data predicted the activation of “nervous system development” and “axon guidance” among the main activated pathways (Figure 1B).

The upregulation of neurotrophic genes in Itga7^+^ cells after denervation suggests that this population includes cell types endowed with potential nerve repair activities. Therefore, to identify this population, we sought to determine whether the activation of the neurotrophic gene program also occurred in Itga7^+^ cells isolated from muscles subjected to sciatic nerve crush — a reversible lesion that is typically followed by repair and restoration of NMJ integrity (21). Indeed, the upregulation of neurotrophic genes was also detected by quantitative PCR (qPCR) analysis in Itga7^+^ cells following sciatic nerve crush (Figure 1C). Interestingly, activation of the same genes was also observed in cells isolated using the Satellite Cell Isolation Kit (Miltenyi Biotec) magnetic strategy that is based on lineage marker exclusion of Itga7^–^ muscle-resident cells (Supplemental Figure 1A; supplemental material available online with this article; https://doi.org/10.1172/jci.insight.143469DS1).

Given the recent studies showing that Itga7-expressing cell populations in muscle include cellular subsets divergent in developmental origin from the actual MuSCs (22), we used the PAX7^CreER^ tdTomato*^fl/fl^* mouse model, which allows MuSCs lineage tracing (23), in order to unequivocally determine whether the activation of the neurotrophic gene program occurs in MuSCs or the non-MuSC cell fraction among the Itga7^+^ cells. In this mouse model, tamoxifen (Tmx) treatment leads to the permanent expression of tomato fluorescent protein in Pax7^+^ MuSCs (Supplemental Figure 1B). Seven days after Tmx treatment, 3-month-old mice were subjected to nerve injury and analyzed 3 days later (Figure 1D). As expected, Itga7^+^Tomato^+^ cells isolated from limb muscles of uninjured mice or following sciatic nerve crush expressed high level of the MuSC-identity genes Pax7 and Vcam1, whereas the Itga7^+^Tomato^–^ cell population did not express these markers (Figure 1E). However, upon denervation, only the Itga7^+^Tomato^–^ fraction expressed Ngfr, Shh, Tnc, Nrcam, and Gdnf, indicating the induction of a neurotrophic signaling pathway in the Pax7^–^ fraction of Itga7^+^ cells (Figure 1E and Supplemental Figure 1C). These observations also indicate that among the cells isolated with the Satellite Cell Isolation Kit (Miltenyi Biotec), nonmyogenic Itga7^+^ and Pax7^–^ cell types might also cosegregate with the myogenic fraction.

Overall, these findings show transcriptional activation of a neurotrophic program in the nonmyogenic fraction of Itga7^+^ cells and suggest their potential involvement in the functional crosstalk between muscle-resident cells and NMJ in response to nerve injury.

### Single-cell RNA-Seq reveals heterogeneity within the Itga7^+^ muscle-resident cells.

**Figure 2:**
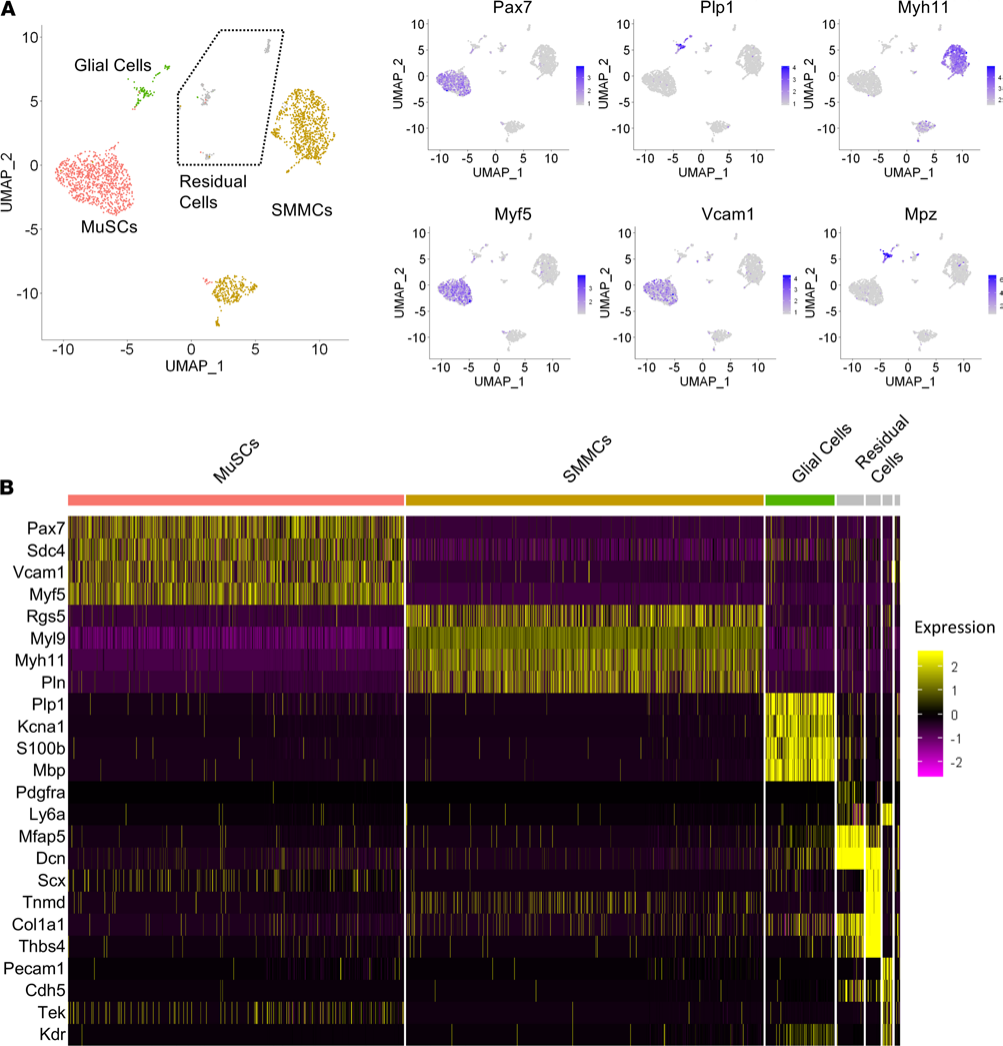
Itga7^+^ cell heterogeneity revealed by single-cell RNA-Seq analysis. (**A**) Distribution of Pax7, Plp1, Myh11, Myf5, Vcam1, and Mpz transcripts in Uniform Manifold Approximation and Projection–derived (UMAP-derived) clusters of single cells (scRNA-Seq) of ltga7^+^Sca1^–^Ln^–^ isolated cells from control muscle. (**B**) RNA expression heatmap for the given cell populations (column) and genes (row), sorted by clusters. The canonical markers used to identify each cluster are plotted (or the most variable genes per cluster in cases where markers were not already present in the literature). MuSCs, muscle satellite cells; SMMCs, smooth muscle mesenchymal cells.

Recently, the heterogeneous composition of muscle-resident mononuclear cells has been dissected using high resolution cartography by single-cell RNA-Seq (scRNA-Seq). This strategy has revealed the identity of a new MuSCs-independent myogenic population among the Itga7^+^ cells, referred to as smooth muscle mesenchymal cells (SMMCs) (22). To further determine whether the activation of the neurotrophic program upon nerve injury occurred in SMMCs or in other subpopulations within Itga7^+^ cells, we performed scRNA-Seq transcriptome profiling in Itga7^+^ cells isolated from limb muscles of mice 3 days following sciatic nerve crush or control mice. By using the 10× Genomics’ scRNA-Seq technology, we obtained a total of 3949 cells analyzed. Clustering analysis identified 11 different groups (Supplemental Figure 2A). Based on markers expression we could clearly identify 3 major populations that were composed of multiple subclusters (Figure 2, A and B). In particular, we detected MuSCs, as Pax7^+^-, Myf5^+^-, and Vcam1^+^-expressing cells, SMMCs, as Myh11^+^-expressing cells, and glial cells as Plp1- and Mpz-expressing cells (Figure 2A).

Interestingly, within the SMMC population we could distinguish a Myl9^+^Rgs5^lo^ and Myl9^+^Rgs5^hi^ subpopulations (Supplemental Figure 2B). In line with this observation, Rgs5, Myl9, and Plp1 expression was evident in the tomato^–^ fraction of Tmx-treated PAX7.Cre_tdTomato mice (Supplemental Figure 2C). The gene expression profile of the smaller clusters (less than 150 cells) was indicative of myotendinous (Scx^hi^-expressing cells), endothelial (Pecam1^hi^), and mesenchymal (as Ly6a/Ly6e^pos^ and Pdgfra^pos^) lineages (Figure 2B). However, it is likely that the presence of these clusters is attributed to contamination from Itga7^lo/neg^ populations.

### Plp1^+^ glial cells are the major Itga7^+^ population responsive to denervation.

**Figure 3:**
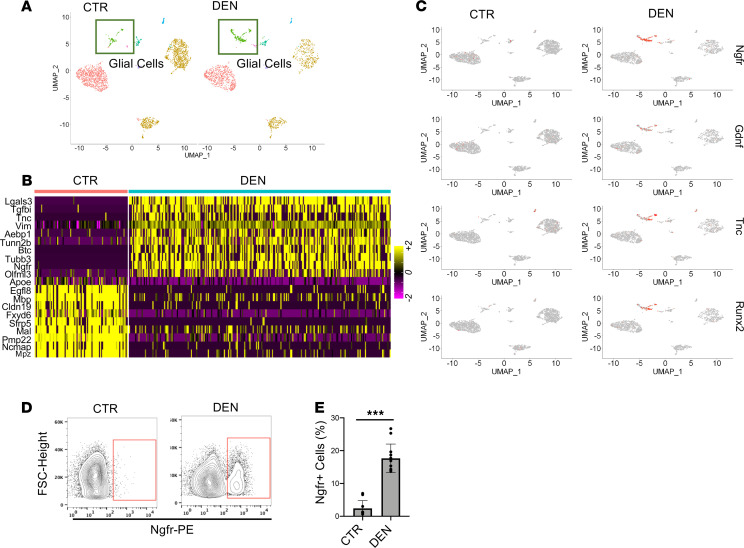
Activation of a neurotrophic signaling pathway in muscle glial cells upon denervation. (**A**) Distribution in UMAP-derived clusters of single cells (scRNA-Seq) of ltga7^+^Sca1^–^Ln^–^ isolated cells from control (CTR, left) and 3-days denervated muscle (DEN, right). (**B**) RNA expression heatmap for Plp1 cell populations isolated from control and denervated muscle (column) and genes (row), sorted by clusters. (**C**) Distribution of Ngfr, Gdnf, Tnc, and Runx2 transcripts in UMAP-derived clusters of single cells (scRNA-Seq) of ltga7^+^Sca1^–^Ln^–^ isolated cells from control (left) and denervated (right) muscle. (**D**) Representative cytofluorimetric plot of Ngfr^+^— gated within the ltga7^+^Sca1^–^Ln^–^ population — cells in control (left) and denervated (right) muscle. (**E**) Quantification of Ngfr^+^ cells is shown in the graphs as a percentage of ltga7^+^Sca1^–^Ln^–^ population (*n* = 8 CTR, *n* = 10 DEN, values represent mean ± SD, ***P < 0.001; by Mann-Whitney test).

We focused our analysis on the 3 major cell types identified by scRNA-Seq analysis among the Itga7^+^ cells isolated following nerve injury, i.e., MuSCs, SMMCs, and glial cells. We observed a similar distribution of MuSCs and SMMCs in both healthy and denervated muscle (Figure 3A). By contrast, we observed a specific increase in Plp1^+^ cells in response to nerve injury. To validate this finding, we denervated mice by nerve crush injury, and at 12 hours before harvest we administered an i.p. injection of 5-ethynyl-2’-deoxyuridine (EdU). EdU incorporation analysis revealed a significant proportion of proliferating Plp1^+^ cells at 3 days following sciatic nerve crush (Supplemental Figure 3A). The gene expression profile of nerve injury–activated Plp1^+^ cells largely overlapped with those of glial cells recently described by both De Micheli et al. and Giordani et al. (22, 24) (Supplemental Figure 3B); however, there was no significant overlap with other populations (in particular with MuSCs and SMMCs) (Supplemental Figure 3B). In addition, these cells were enriched in additional markers known to be expressed by glial/Schwann cells (Supplemental Figure 3C).

Furthermore, we noted extensive alterations in gene expression in Plp1^+^ cells following nerve injury, compared with control muscle, whereas only a few genes were significantly altered in MuSCs and SMMCs (Supplemental Figure 3D). scRNA-Seq analysis revealed that Plp1^+^ glial cells are characterized by a neurotrophic signature (Figure 3, B and C, and Supplemental Figure 3D), with most of the upregulated genes coinciding with those identified in the bulk Itga7^+^ cells shown in Figure 1, i.e., Ngfr, Tnc, Gdnf, Runx2, and other genes strictly related to nerve development (Figure 3, B and C). Among these genes, Ngfr was used to identify these cells, as it was found to be specifically expressed in glial cells, and no other muscle-resident cells, in response to nerve injury, by an independent scRNA-Seq analysis after denervation (25). It is interesting to note that Hicks et al. have previously shown that Ngfr is transiently expressed in human MuSC progenitors during the generation of hiPSC-derived MuSCs (26). Our data indicate that Ngfr expression discriminates glial cells from MuSCs within a common pool of Itga7^+^ cells. Ngfr^+^ cells were only detected among the Itga7^+^ population in the muscle at 3 days after denervation, whereas only a negligible number of Ngfr^+^ cells were present in control muscle (Figure 3, D and E, and Supplemental Figure 4, A–C). There were no significant changes in the number of Ngfr^–^Itga7^+^ cells after nerve injury (Supplemental Figure 4D), thus confirming that the main cell type responding to denervation are the muscle-resident glial cells. Indeed, the number of Pax7^+^ cells did not change at 3 days after nerve injury, and Pax7 did not colocalize with Ngfr in serial muscle sections (Supplemental Figure 4, E and F). Moreover, the induction of neurotrophic genes, including Ngfr, but also Plp1, Tnc, Nrcam, and Gdnf, was only detected in Ngfr^+^ cells, but not Ngfr^–^ cells, isolated from denervated muscle (Supplemental Figure 4G). Finally, the Ngfr^+^ cells differed in phenotype and morphology from the Ngfr^–^ population, and unlike the Ngfr^–^ cells, they did not show any myogenic potential when cultured in vitro (Supplemental Figure 5, A and B).

Interestingly, the presence of Ngfr^+^ cells was also observed in a mouse model of spinal chord injury (SCI) in which the damage was induced by mechanical lesion of the spinal cord (Supplemental Figure 5, C and D). Moreover, at 7 days after spinal lesion, the appearance of Ngfr^+^ cells was observed in tibialis anterior (TA) muscle. We found that in unperturbed muscles Plp1^+^ cells are close to the neuron structure stained for neurofilament (NF-L) that is lost upon nerve injury. However, upon nerve injury, Plp1^+^/Ngfr^+^ cells were found in a structure reminiscent of the nerve structure and close to bungarotoxin^+^ (BTX^+^) NMJs (Supplemental Figure 4, A–C). Moreover, a decline in the number of the Ngfr^+^ cells and, more importantly, a decrease in neurotrophic expression were observed at 30 days after nerve injury, when the reinnervation process typically occurs (Supplemental Figure 6, A and B).

**Figure 4:**
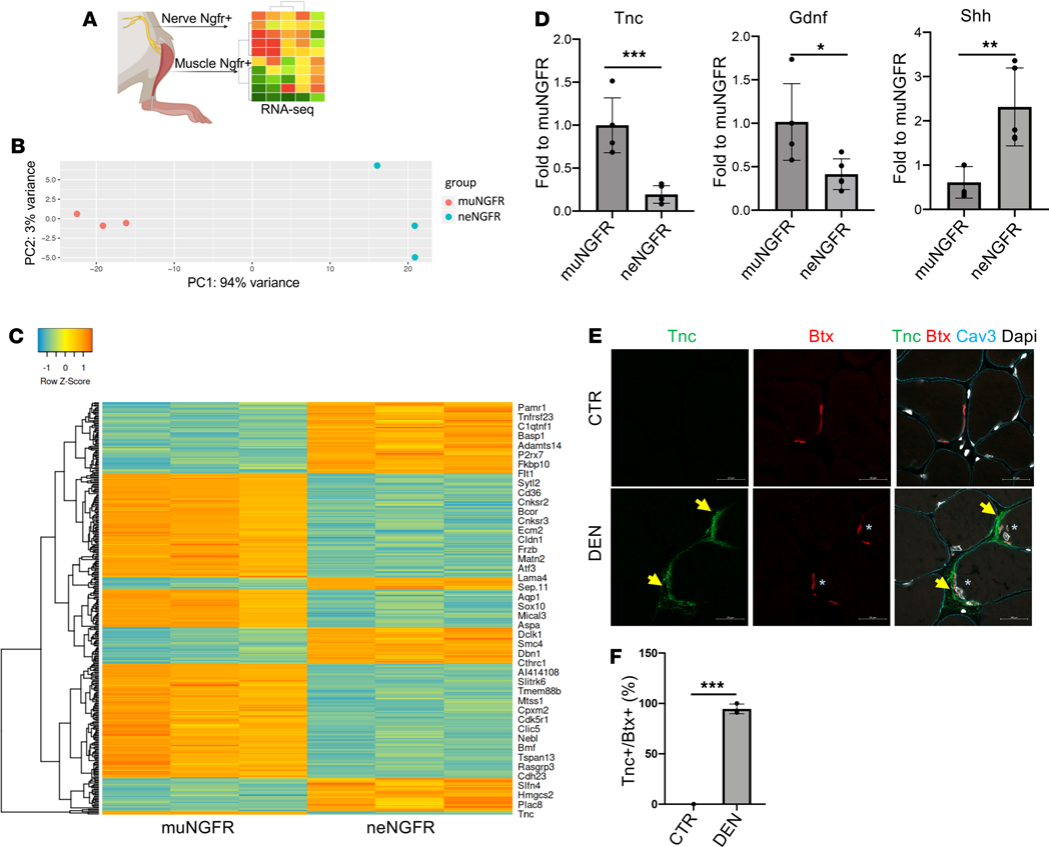
A specific transcriptional signature distinguishes glial cells in muscle from those residing in the nerve. (**A**) Experimental setting for RNA-Seq analysis of Ngfr^+^ cells derived from denervated muscle and nerve at 3 days after nerve lesion. (**B**) Sample distance — represented as principal component analysis (PCA) — of transcriptome of Ngfr^+^ cells derived from denervated muscle and nerve at 3 days after nerve lesion (*n* = 3). (**C**) Heatmap representation of genes significantly deregulated — *P* adj < 0.001 — in freshly isolated Ngfr^+^ cells derived from denervated muscle and nerve at 3 days after nerve lesion (*n* = 3). (**D**) qPCR analysis for Tnc, Gdnf, and Shh expression in freshly isolated Ngfr^+^ cells derived from denervated muscle (muNGFR) and nerve (neNGFR) at 3 days after nerve lesion (*n* = 4, values represent mean ± SD.*P < 0.05, **P < 0.01, ***P < 0.001; by 2-tailed Student’s *t* test Tnc, Gdnf or by Mann-Whitney, Shh). (**E**) Representative immunofluorescence analysis of TA muscle cryosection derived from control and denervated muscle, stained for Tnc (green), Bungarotoxin (Btx, red), and Caveolin-3 (Cav3, Cyan). Arrows highlight Tnc and asterisks highlight Btx. Nuclei were counterstained with DAPI. Scale bar: 20 μm. (**F**) Quantification Btx and Tnc colocalization in control and denervated muscle (*n* = 3, values represent mean ± SD, ***P < 0.001; by 2-tailed Student’s *t* test).

Activated glial cells are known to respond to nerve injury and participate in nerve repair and axon guidance. To exclude the possibility that the presence of activated glial cells within the muscle was simply caused by coisolation of adjacent tissues (i.e., nerves), we compared the gene expression profile of Ngfr^+^ cells isolated from nerves or muscles at 3 days after nerve injury (Figure 4A). Transcriptome analysis revealed significant differences between the 2 populations, which clearly formed 2 independent clusters with a large subset of differentially regulated genes (Figure 4, A–C). Interestingly, among the genes differentially expressed by the 2 glial cell populations we found some genes more represented in muscle-derived glial cells (i.e., Tnc and Gdnf), whereas others are more expressed in neuron-derived cells (i.e., Shh) (Figure 4D). We focused on Tnc since its ablation is known to delay NMJ recovery in vivo (27). Tnc protein unequivocally localized close to BTX^+^ NMJ upon denervation (Figure 4, E and F, and Supplemental Figure 6C). Finally, Tnc protein expression and localization close to the NMJ was observed in TA muscle at 7 days following spinal cord injury (Supplemental Figure 6D). These results suggest an involvement of Tnc in the maintenance of NMJ following denervation.

### Muscle-resident glial cells activated by nerve injury adopt a defective neurotrophic phenotype in a mouse model for ALS.

The symptomatic stage of ALS is characterized by muscle denervation. Motoneuron degeneration leads to muscle atrophy and muscle weakness, ultimately accelerating disease progression (28). It is currently unclear whether the disease progression could be influenced by the neurotrophic activity of specialized cell types. We therefore set to determine whether an increased amount of muscle resident Plp1^+^/Ngfr^+^ glial cells could be observed at sequential stages of disease progression, using the SOD^G93A^ mouse model of ALS (29).

**Figure 5:**
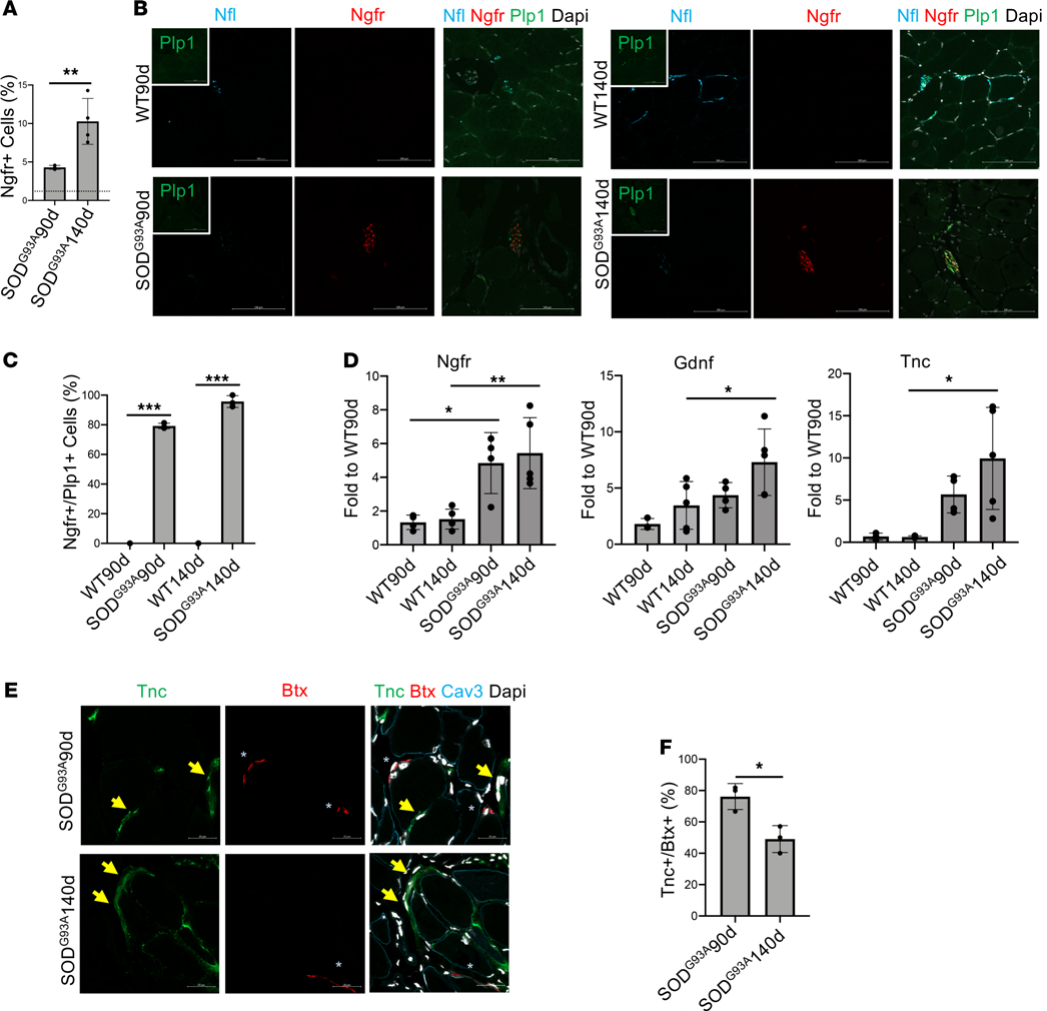
Muscle-resident glial cell activation in a mouse model of ALS. (**A**) Ngfr^+^ cell cytofluorimetric quantification was shown in the graphs as a percentage of Itga7^+^Sca1^–^Ln^–^ population, in 90- and 140-day-old SOD^G93A^ mice muscle (*n* = 4, values represent mean ± SD. **P < 0.01; by 1-way ANOVA Tukey’s multiple-comparison test). Dotted line highlights percentage in WT mice muscle. (**B**) Representative immunofluorescence analysis of TA muscle cryosection derived from 90- and 140-day-old SOD^G93A^ and WT mice stained for neurofilament-L (Nfl, Cyan), Ngfr (red), and Plp1 (green). Nuclei were counterstained with DAPI. Scale bars: 100 μm. (**C**) Quantification graph of Ngfr^+^/Plp1^+^ cells in 90- and 140-day-old WT and SOD^G93A^ mice muscle (*n* = 3, values represent mean ± SD; ***P < 0.001; by 1-way ANOVA Tukey’s multiple-comparison test). (**D**) qPCR analysis for Ngfr, Gdnf, and Tnc expression in freshly isolated Itga7^+^Sca1^–^Ln^–^ cells derived from WT and SOD^G93A^ muscle at 90 and 140 days of postnatal life. GAPDH was used as housekeeping gene (*n* = 4, values represent mean ± SD; *P < 0.05, **P < 0.01; by 1-way ANOVA Tukey’s multiple-comparison test). (**E**) Representative immunofluorescence analysis of TA muscle cryosection derived from 90- and 140-day-old SOD^G93A^ and WT mice stained for Tnc (green), Btx (red), and Caveolin-3 (Cav3, cyan). Arrows highlight Tnc and asterisk highlights Btx. Nuclei were counterstained with DAPI. Scale bar: 20 μm. (**F**) Quantification of Btx and Tnc colocalization in 90- and 140-day-old SOD^G93A^ mice muscle (*n* = 3, values represent mean ± SD *P < 0.05; by 2-tailed Student’s *t* test).

Ngfr expression and Ngfr^+^ cells among Itga7^+^ cells increased with disease progression (Figure 5, A–D). Ngfr expression displayed a striking increase in muscle derived from symptomatic SOD^G93A^ at 90 and 140 days of postnatal life with a concomitant reduction of NF-L^+^ positive neurofilament in accordance with the progressive loss of muscle innervation (Figure 5, B–D). Other factors such as Gdnf or Tnc also increased significantly in SOD^G93A^ in the late stage of the disease (140 days) compared with age-matched healthy animals (Figure 5D). Overall, these observations clearly identify muscle glial cells as potential players in the maintenance of nerve-to-muscle contact in the context of ALS. Interestingly, the magnitude of the induction of Tnc and Gdnf was clearly lower compared with acute denervation (Figure 1). This may account for the reduced reinnervation ability of SOD^G93A^ leading to muscle paralysis that marks the end stage of the disease. In addition to lower Tnc expression compared with reversible nerve degeneration, we also observed a marked difference in Tnc localization in ALS muscle. Although in reversible denervation we observed a clear localization of Tnc protein close to the NMJ, in SOD^G93A^ muscle Tnc signal encircled muscle fibers without the NMJ-associated pattern (Figure 5, E and F). These data suggest a defect in the response of glial cells during disease progression.

**Figure 6:**
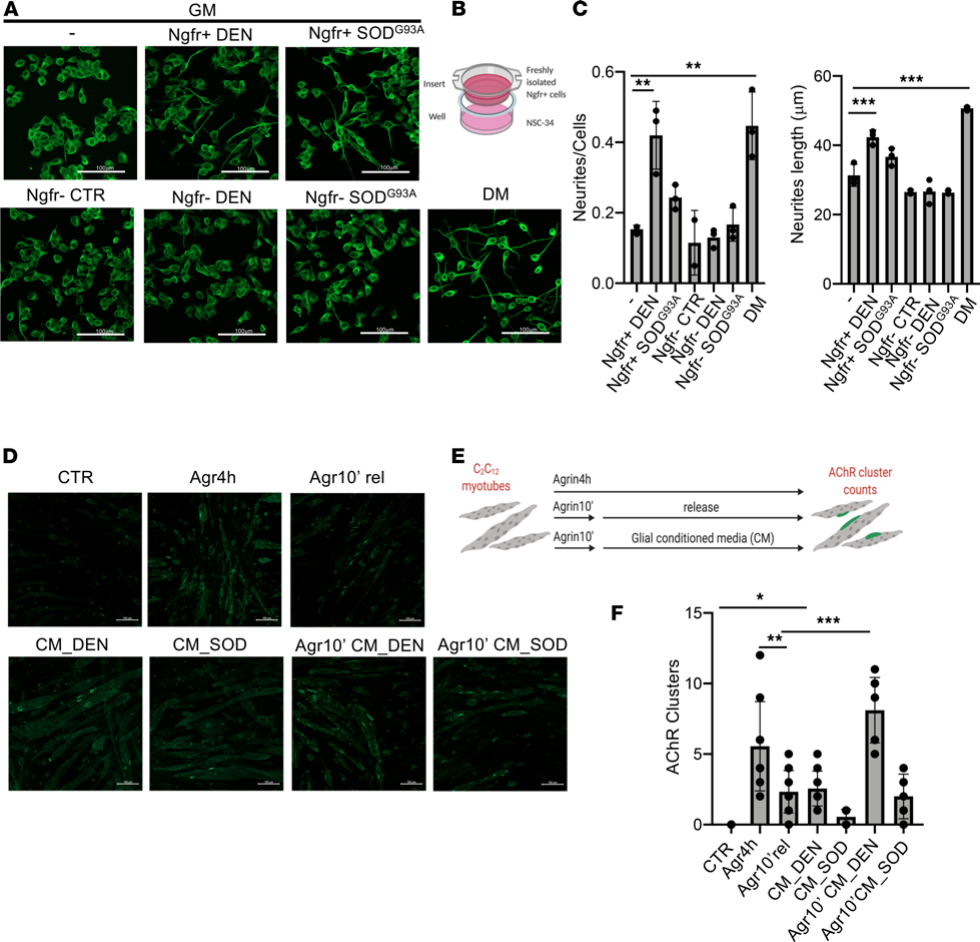
Muscle-resident glial cells promote neurite outgrowth and AChR clustering. (**A**) Representative immunofluorescence analysis of NSC-34 cells in growth media cultured either alone (-) or in coculture with Ngfr^+^ or with Ngfr^–-^ cells, both from denervated muscle and SOD^G93A^ muscle at 90 days of postnatal life, and of NSC-34 cells cultured in neurogenic differentiation media (DM), stained for β-3-Tubulin (green). Scale bars: 100 μm. (**B**) Schematic representation of in vitro coculture system. (**C**) Quantification of neurite number per cell and length of NSC-34 cultured in the indicated conditions (*n* = 3, values represent mean ± SD; **P < 0.01, **P < 0.01; by 1-way ANOVA Tukey’s multiple-comparison test). (**D**) Representative immunofluorescence analysis of C2C12 myotubes treated or not with agrin or conditioned media from glial cells as indicated and stained with Btx (green). Scale bars: = 100 μm. (**E**) Schematic representation of the experimental setting. (**F**) Quantification of AChR clustering (25 μm) (*n* = 5, values represent mean ± SD; *P < 0.05, **P < 0.01, ***P < 0.001; by 1-way ANOVA Tukey’s multiple-comparison test).

To functionally validate the neurotrophic ability of Plp1^+^Ngfr^+^ muscle-derived glial cells, we used an in vitro Transwell system (Figure 6, A–C). Ngfr^+^ and Ngfr^–^ cells were isolated from limb muscle of mice subjected to nerve injury or from symptomatic SOD^G93A^, and cocultured with the mouse motor neuron–like hybrid cell line (NSC-34), without direct contact through the use of specific Transwell inserts. Following 72 hours of coculture in growth media, Ngfr^+^ cells from denervated muscle promoted NSC-34 neuronal differentiation when compared with control cells cultured under standard neuronal differentiation conditions, as documented by the increase in neurite length and the mean number of neurites per cell (Figure 6, A–C). Conversely, this effect was not observed in NSC-34 cocultured with Ngfr^–^ cells. Interestingly, a lower ability to promote NSC-34 differentiation and neurite elongation was observed in cells cocultured with Ngfr^+^ cells derived from SOD^G93A^ muscle (Figure 6, A–C).

These data suggest that an impaired ability of muscle-resident glial cells to adopt a neurotrophic phenotype in response to nerve injury could contribute to progressive loss of NMJ in ALS muscles. Next, given the localization of glial cells close to the NMJ, we explored the possibility that they may play a direct role in the promotion and maintenance of NMJ upon denervation. We tested this possibility by using a model of acetylcholine receptor (AChR) clustering in cultured myotubes, previously described by Ngo and colleagues (30). As shown in Figure 6, D–F, and Supplemental Figure 7, conditioned media from muscle-derived glial cells promote AChR clustering — as revealed by BTX staining — in differentiated C2C12 myotubes. As a positive control, a 4-hour agrin (Agr) treatment was used. Interestingly, whereas after a 10’ pulse of Agr followed by 4 hours of release the AChR clustering was reduced, as previously shown (30), replacement of conditioned media after Agr pulse led to a similar induction of AChR clustering compared with a full 4-hour Agr treatment. These observations support a direct role of muscle glial–released factors in the induction and maintenance of the muscle counterpart of the NMJ. Notably, we observed that the formation of AChR clustering was attenuated when using media conditioned by glial cells isolated from the SOD^G93A^ muscle, further suggesting a functional impairment of muscle glial cell function during ALS progression.

## Discussion

scRNA-Seq–based analysis has been instrumental to unravel the heterogeneity of muscle-resident cells in unperturbed conditions, while highlighting their dynamic transitions through a continuum of functional cellular states and trajectories in response to homeostatic perturbations (22, 24, 31–37). These studies have used the typical experimental model of muscle regeneration, by physical injury, which leads to the sequential activation of multiple cell types, to reveal the identity of subpopulations endowed with specialized activities and their coordination in response to regeneration cues. In addition to MuSC activation, scRNA-Seq analyses has revealed a dramatic expansion and alteration of gene expression profiles in cells from the inflammatory infiltrate immediately after acute muscle injury (24, 34, 37). These cells account for the vast majority of the cell types present in muscles at early time points after injury and establish functional interactions with other cell types within the regenerative environment, including FAPs and MuSCs, to promote myofiber regeneration and injury resolution. As part of the regeneration process, the repair of injured nerve also occurs, although the cellular effectors of this process remain poorly understood. Although earlier studies have suggested the potential contribution of MuSCs in the maintenance of NMJ integrity and regeneration (9, 10), the precise identity of the cell types activated by nerve injury and their potential neurotrophic activity remain unknown.

Unlike muscle injury, nerve injury does not promote muscle regeneration, but leads to myofiber atrophy and muscle fibrosis (11, 38). These different outcomes are underpinned by differences in activated cell types. For instance, we have previously observed that muscle denervation leads to the selective activation of FAPs, which exhibit transcriptional profiles and biological activities different from FAPs activated in response to acute muscle injury (11, 31). Importantly, denervation does not trigger the massive infiltration of immune cells observed upon muscle injury (11). Therefore, we argue that the lack of inflammatory infiltrate and the consequent reduction in the number of inflammatory signals in the milieu of denervated muscles might account for the lack of activation of multiple cell types in denervated muscles (25). At the same time, the selective response of muscles to denervation might help to capture specific muscle-resident cells activated by nerve injury, without the potentially confounding coexistence of other activated cell types.

In this study, we describe a population of muscle-resident glial cells that are activated by nerve injury and might contribute to NMJ repair. These cells express Itga7 — a cell surface protein commonly used to prospectively isolate MuSCs (39). Indeed, both previously used FACS strategies and commercial kits could not distinguish the 2 populations owing to the common antigen surface marker. A recent study identified a population of SMMCs within the FACS-isolated Itga7^+^ cells that is distinct from MuSCs (22). Interestingly, our results indicate that muscle-resident glial cells, although sharing Itga7 expression with MuSCs and SMMCs, exhibit a distinctive gene expression signature that is enriched in glial cell–specific genes. Although some glial lineage–identity marker was constitutively expressed and could be used for their prospective isolation in unperturbed muscles (Plp1), a subset of neurotrophic genes was selectively expressed in these cells only in response to nerve injury. Among them, Ngfr was instrumental to further isolate the fraction of Itga7/Plp1 glial cells activated upon nerve injury (Figure 3). Ngfr is a receptor commonly associated with activated glial cells (40). Since Ngfr expression in Itga7^+^ muscle glial cells is only observed upon their activation by nerve injury and coincides with the activation of the neurogenic program, it is conceivable that Ngfr confers upon glial cells the competence to respond to neurogenic signals.

Although nerve-associated glial cells (otherwise defined as Schwann cells) are well known, the biological properties of tissue-resident glial cells have only recently become the object of intense investigation (41).

In the case of skeletal muscle, the specific function of peripheral glial cells and their regulation in response to homeostatic perturbations, such as in ALS disease, are currently not well known (19, 42). Although several myelinating and nonmyelinating cell types (Remak and terminal Schwann cells) have been associated with neuron regeneration, a defined molecular signature able to discriminate between subpopulations from different anatomical location and different functional specialization has not been clearly identified. Indeed, the molecular features of terminal Schwann cells remain mostly unknown, because their scarcity has so far impeded a comprehensive analysis (43). Our data indicate that scRNA-Seq–based approaches may circumvent this issue owing to their potential to identify transcriptional signatures in a small number of cells within the pool of cells analyzed.

Our work identified a population of Itga7-expressing cells, distinct from MuSCs and SMMC, that is selectively activated upon nerve injury and adopt a neurogenic gene expression profile and functional neurotrophic properties. Interestingly, activated muscle-resident glial cells localize in close proximity to NMJs. Although a comparative analysis using the scRNA-Seq profiles of Ngfr^+^ cells isolated either from nerves or from muscles at 3 days after nerve injury revealed clear differences in gene expression between these 2 populations, it is possible that they might represent 2 different functional states of muscle-resident glial cells. Nevertheless, the differential expression of certain genes, such as Tnc, Gdnf, and Shh, suggests that muscle-resident glial cells adopt different functional phenotypes in response to nerve injury.

The upregulation of Tnc in muscle-resident glial cells activated by nerve injury has not been shown previously, and highlights a fundamental difference between the skeletal muscle response to nerve injury versus myotrauma. In the latter, Tnc is typically expressed by other types of resident-muscle cells (e.g., FAPs) and accumulates within the extracellular matrix (ECM) to regulate MuSC activity (23, 24, 34, 37). Conversely, we show that in response to nerve injury, Tnc accumulates in close proximity to BTX^+^ NMJ. Thus, higher levels of Tnc in muscle-derived glial cells and its anatomical localization in proximity of NMJ are distinctive features of skeletal muscle response to NMJ injury. Considering that genetic ablation of Tnc causes delay in NMJ recovery in vivo (27), we speculate that muscle glial cell–derived Tnc could contribute to NMJ repair following injury. This possibility is also supported by the finding that Tnc expression was reduced upon recovery of NMJ integrity (e.g., up to 30 days after lesion). It is possible that transient deposition of Tnc within the ECM at NMJ is an important event to promote NMJ repair and is part of a general program by which muscle glial cells commit to repair injured muscles. Consistently, we found that factors secreted by muscle glial cells could enhance AChR clustering in cultured myotubes.

Further studies will be necessary to investigate the actual contribution of muscle glial cells in the recovery of NMJ integrity in response to acute lesions or chronic degeneration, and whether these cells might be amenable to pharmacological manipulation to facilitate nerve repair. In this regard, pharmacological activation of the neurotrophic potential of muscle glial cells could be exploited in neurodegenerative disorders, such as ALS. A role for peripheral glial cells in ALS — and in particular presynaptic Schwann cells — has been recently suggested, although the precise mechanism of their involvement remains unknown (42, 44). We found a progressive increase of muscle-resident glial cells in muscles of the ALS mouse model — SOD^G93A^ mice. However, activated muscle glial cells from symptomatic SOD^G93A^ mice exhibited reduced activation of neurotrophic genes, defective Tnc localization, impaired ability to promote neurite outgrowth/differentiation of a motoneuron cell line, and to promote AChR clustering in cultured myotubes, as compared with glial cells activated in the context of acute reversible denervation. These data suggest that defective activity of muscle glial cells could contribute to the pathogenesis of neurodegenerative diseases, such as ALS.

## Methods

### Mouse strains.

C57BL/6J mice were provided by The Jackson Laboratory. PAX7CreER/tdTomato*^fl/fl^* mice were provided by the SBP Animal Facility. Hemizygous transgenic mice carrying the mutant human SOD1^G93A^(B6.Cg-Tg(SOD1*G93A)1Gur/J) gene (referred to herein as SOD mice) were originally obtained from The Jackson Laboratory. CD1 mice were provided by Charles River Laboratories.

All mice were maintained in a pathogen-free animal facility under a standard 12-hour light/12-hour dark cycle at 21°C with access to red house and to standard chow and water ad libitum. Three-month-old mice were used for ex vivo experiments, except for the SOD^G93A^ mice, as shown in Figure 5. For the denervation experiments, both male and female C57BL/6J and PAX7CreER/tdTomato^fl/fl^ mice were used. Only female mice were used for the spinal cord injury experiment, as a mouse model of ALS male SOD^G93A^ mice were used.

### Cell lines and primary cell cultures.

All cells were cultured in incubators at 37°C and 5% CO_2_. We used mouse motor neuron–like NSC-34 cells (obtained from ATCC), which is a hybrid cell line produced by the fusion of MNs from the spinal cord embryos with N18TG2 neuroblastoma cells that exhibit properties of MNs after differentiation and maturation protocols (45). Thus, NSC-34 cells were grown in proliferation media (DMEM:Nutrient Mixture F-12 [DMEM/F-12, MilliporeSigma, D6421] supplemented with 10% FBS [MilliporeSigma, F4135] and 1% penicillin/streptomycin [Gibco, 15070-063]). Differentiation was induced by changing medium for DMEM/F-12 plus 0.5% FBS, 1% nonessential amino acids (NEAA, Thermo Fisher Scientific, 11140050), and 1% penicillin/streptomycin.

Freshly isolated MuSCs and Ngfr^+^ cells were plated in 24-well plates in GM (DMEM [+Pyruvate], Gibco, 41966-029; 20% FBS; 10% horse serum; and 1% chick embryo extract [CEE]). Myogenic differentiation was induced with DMEM and 2% horse serum for 2 or 3 days. NSC-34 and Ngfr^+^ cells were also used for coculture experiments.

C2.12 (C2C12) myogenic cells were obtained from ATCC and cultured on 96-well plates in growth medium (DMEM [-Pyruvate], Gibco, 61965-026; supplemented with 10% of FBS and 1% of penicillin/streptomycin). Myogenic differentiation was induced by shifting the cells in differentiation medium (DMEM [-Pyruvate] complemented with 1% penicillin/streptomycin and 2% horse serum).

### AChR clustering assay.

AChRs were considered to be a large AChR cluster when they were equal to or more than 25 μm in their longest dimension. C2C12 myotubes were treated with 1 nM recombinant rat Agr (R&D System, 550-AG) for 4 hours or for 10 minutes in differentiation medium (30). Different C2C12 myotube cultures were treated with conditioned media of muscle-derived glial cells for 4 hours or after the 10-minute pulse of Agr. AChRs were labeled by the binding of Alexa Fluor 488 α-BTX (Invitrogen, B13422). Myotubes were incubated with the α-BTX diluted (1:300) in differentiation medium for 1 hour at 37°C in 5% CO_2_. The number of AchR clusters per field in their longest dimension (≥25 μm) was measured using ImageJ software (NIH).

### Denervation.

Unilateral hindlimb denervation was performed by clamping the left sciatic nerve under anesthesia by i.p. injection of 40 mg/kg ketamine (Zoletil, Virbac) and 10 mg/kg xylazine (Rampum, BAYER). Upon exposure of the sciatic nerve, the nerve was crushed 3 times for 10 seconds. Alternatively, as shown in Figure 1A, for the bulk RNA-Seq, the nerve was cut with a scissor. The lesion was sutured after the operation.

### Spinal cord injury.

Three-month-old CD1 mice were used in SCI. To perform SCI, mice were deeply anesthetized with a mixture 1:1 of Rompun (Bayer, 20 mg/mL; 0.5 mL/kg) and Zoletil (100 mg/mL; 0.5 mL/kg), the back hairs were shaved, the skin was disinfected with betadine, and an incision was made to expose the spinal cord. Animals were mounted on a stereotaxic apparatus with spinal adaptors connected to a cortical PinPoint precision impactor device (Stoelting) and maintained at 37°C throughout surgery. To induce a severe trauma, the following parameters were set up: middle, round, and flat tip (#4); velocity, 3 m/s; depth, 5 mm; and dwell time, 800 ms. The impact was applied at the thoracic level (vertebrae T10–T11). Analysis of the graphical impact parameters, operated by the PinPoint software, was used to identify potential outliers. Behavioral analyses were also used to corroborate differences in injury severity within groups. Slight lesions were excluded from the study based on these criteria.

### Cell preparation and isolation by FACS.

TA and gastrocnemius muscles, or nerves, of mice were subjected to enzymatic dissociation (in PBS with 2 mg/mL Collagenase A [Roche, 10 103 586 001], 2.4 units/mL Dispase I [Roche, 04 942 078 001], 10 ng/mL DNase [MilliporeSigma, 11 284 932 001], 0.4 mM CaCl_2_ and 5 mM MgCl_2_) for 60 minutes at 37°C. The cell suspension was filtered through a 40 μm nylon filter and incubated with the following antibodies for 30 minutes: CD45 (Invitrogen, 48-0451-82), CD31 (Invitrogen, 48-0311-82), TER119 (Invitrogen, 48-5921-82), Sca1 (Invitrogen, 11-5981-82) and Itga7 (AbLab, R2F2), and Ngfr (Miltenyi Biotec, 130-118-793).

Ngfr^+^ cells were isolated as TER119^−^CD45^−^CD31^−^Itga7^+^SCA-1^−^Ngfr^+^ cells and Pax7^–^Tomato^+^ as TER119^−^CD45^−^CD31^−^Itga7^+^SCA-1^−^Tomato^+^ (Supplemental Figure 1B).

In Supplemental Figure 1A, satellite cell purification was performed by using the SC Isolation Kit (Miltenyi Biotech, 130-104-268).

### Histology immunofluorescence.

For the histological analysis 8 μm muscle cryosection were analyzed. Both cryosections and cultured cells were fixed in 4% PFA (MilliporeSigma, P6148) for 10 minutes and permeabilized with 100% acetone for 1 minute at room temperature or with 0.1% Triton X-100 for 15 minutes at room temperature. Muscle sections and cultured cells were then blocked for 1 hour with a solution containing 4% BSA (MilliporeSigma, A7030-100G) in PBS. PAX7 staining was performed by an antigen retrieval protocol. The primary antibody immunostaining was performed overnight at 4°C, and then the antibody binding specificity was revealed using secondary antibodies coupled to Alexa Fluor 488, 594, or 647 (Invitrogen, goat anti-mouse Alexa Fluor 647 [1:400, A32728], goat anti-rabbit Alexa Fluor 488 [1:400, A32731], goat anti-mouse Alexa Fluor 488 [1:400, A32723]). AChRs were revealed with fluorescently labeled BTX (1:500 Alexa 594, Invitrogen, B13423). Sections were incubated with DAPI (Thermo Fisher Scientific, D1306) in PBS for 5 minutes for nuclear staining, washed in PBS, and mounted with glycerol (3:1 in PBS).

The primary antibodies used for immunofluorescences are: rabbit anti-Plp1 (1:100, Cell Signaling, 28702S); mouse anti-NFl (1:200, Santa Cruz Biotechnology, SC-20012); rat anti-Ngfr-PE (1:100, Miltenyi Biotec, 130-118-793); rabbit anti-Tnc (1:100, EMD Millipore Corp., AB19013); mouse anti-Caveolin-3 (1:1000, BD Transduction Laboratories, 610420); rabbit anti-Laminin (1:400, MilliporeSigma, L9393); anti-BIII Tubulin mAb (1:500, Promega, G712A); mouse anti-PAX7 (1:20, Developmental Studies Hybridoma Bank [DSHB], Pax7); mouse anti-Myosin (1:10, DSHB, MF20); and mouse α-Tubulin (1:200, Cell Signaling, 2144).

The transverse sections and cultured cells were visualized on a Zeiss confocal microscope then edited using the ImageJ software. All histological analyses were performed in a blinded fashion. The figures reported are representative of all the examined fields.

### EdU proliferation assay.

Cell proliferation was measured by EdU incorporation. A total of 20 mg per kg body weight EdU was administered i.p. 12 hours before muscle harvest. Incorporation of EdU was revealed using the Click-iT EdU Cell Proliferation Kit for Imaging, Alexa Fluor 594 dye (Thermo Fisher Scientific, C10354) following the manufacture protocol.

### Coculture conditions of Ngfr^+^ and NSC-34 cells.

NSC-34 and Ngfr^+^ cells were cocultured by using inserts with 1 μm porous membrane to avoid direct contact between populations. NSC-34 were grown independently from Ngfr^+^ in proliferation media for 48 hours in 24-well plates. After 24 hours, freshly sorted Ngfr^+^ cells were plated on the upper insert, and Transwell cocultures were maintained for additional 72 hours in proliferation and differentiation media.

### RNA analysis by qPCR.

RNA was extracted from cells using RNeasy Mini kit (Qiagen, 74106) following the manufacturer’s protocol. Total RNA was quantified with a Nanodrop 8000 spectrophotometer (Thermo Fisher Scientific). First-strand cDNA was synthesized from total RNA using the Transcriptor First Strand cDNA Synthesis kit (Roche, N808-0234) following the manufacturer’s protocols. The generated cDNA was used as a template in real-time PCR reactions with 2× Fast Q-PCR Master Mix (SYBR, ROX) (SMOBIO, TQ1211) and was run on a Roche LC480 machine using 3-step amplification and melt curve analysis. Real-time qPCR reactions consisted of 2× SYBR Green Supermix, 0.25 μmol/L forward and reverse primers, and 10 ng cDNA. Relative gene expression was normalized by dividing the specific expression value by the Gapdh expression value and calculated using the 2^−δΔCT^ method.

The following primer sets were used to identify transcripts: Gapdh, forward, CACCATCTTCCAGGAGCGAG, and reverse, CCTTCTCCATGGTGGTGAAGAC; Ngfr, forward, TGCCTGGACAGTGTTACGTT, and reverse, ACAGGGAGCGGACATACTCT; Shh, forward, CACCCCCAATTACAACCCCG, and reverse, CTTGTCTTTGCACCTCTGAGTC; Fgf5, forward, CTGTACTGCAGAGTGGGCAT, and reverse, AATTTGGCTTAACACACTGGC; Runx2, forward, GCCTTCAAGGTTGTAGCCCT, and reverse, GTTCTCATCATTCCCGGCCA; Olig1, forward, CTCGCCCAGGTGTTTTGTTG, and reverse, TAAGTCCGAACACCGATGGC; Tnc, forward, CTACCACAGAGGCCTTGCC, and reverse, AGCAGCTTCCCAGAATCCAC; Pax7, forward, AGGACGACGAGGAAGGAGACA, and reverse, TCATCCAGACGGTTCCCTTT; Vcam1, forward, GCACTCTACTGCGCATCTT, and reverse, CACCAGACTGTACGATCCT; Nrcam, forward, ATGCACAGACATCAGTGGGG, and reverse, GCTTGCCATTGCCTTCTTACC; Gdnf, forward, TGGGTCTCCTGGATGGGATT, and reverse, CGGCGGCACCTCGGAT; Rgs5, forward, CGCACTCATGCCTGGAAAG, and reverse, TGAAGCTGGCAAATCCATAGC; Myl9, forward, GCGCCGAGGACTTTTCTTCT, and reverse, CCTCGTGGATGAAGCCTGAG; and Plp1, forward, CCTAGCAAGACCTCTGCCAGTA, and reverse, GGACAGAAGGTTGGAGCCACAA.

### Tmx treatment and denervation.

We used PAX7CreER/tdTomato^fl/fl^ mice between the ages of 2 and 3 months for Tmx (MilliporeSigma, T5648) injections. Tmx (3 mg) suspended in corn oil was injected i.p. each day for 5 days. After 7 days from the last injection, we performed the unilateral hindlimb denervation. Tissues were harvested after 3 days for FACS.

### RNA-Seq.

MuSCs were isolated from mice TA and GA muscle as described. RNA from MuSC was extracted using RNeasy Mini kit (Qiagen) following the manufacturer’s protocol. RNA was shipped to the sequencing IGA of Udine. The libraries for sequencing were prepared using NuGEN Ovation System V2 RNA-Seq. For each biological sample, 2 independent experiments were carried out for the isolation of RNA. All duplicates are a pool of 3 different mice, sorted at different times.

### RNA-Seq data processing.

For sequencing alignment, we used the mouse reference genome assembly GRCm38/mm10 (http://ftp.ensembl.org/pub/release-76/fasta/mus_musculus/dna/), and for transcriptome annotation, we used version 85 of the GRCm38 (http://ftp.ensembl.org/pub/release-85/gtf/mus_musculus/). We used the FASTQC package (v0.11.3) to assess the quality of sequenced libraries, all of which passed quality control. Reads were mapped to the reference genome using TopHat2 v.2.1.1. The quality control of the reads distribution along transcripts was performed using infer_experiment.py from RSeQC package v2.6.3. All samples had a uniform distribution of reads along transcripts. The sequenced read counts per annotated gene were derived with the use of htseq-count script distributed with HTSeq v0.5.4p5. We used the R library package DESeq2 v.1.12.4 for measuring differential gene expression between 2 different cell conditions, considering the 2 RNA-Seq experiments as biological replicates. We picked genes with an adjusted *P* value of less than 0.001. Gene ontology analysis was performed using David 6.8 (https://david.ncifcrf.gov/). Biological Process was predicted on genes differentially expressed with an adjusted *P* value of less than 0.01 with the following setting: threshold counts = 2, threshold EASE = 0.1. The most significant 12 functional annotation was illustrated in the figure.

### scRNA-Seq.

scRNA-Seq was performed at The Institute of Applied Genomics (IGA) Facility of Udine, Italy (https://igatechnology.com/). Methanol-fixed cells were rehydrated following 10X Genomics recommendation. To remove visible debris, an additional washing in Wash-Resuspension buffer was introduced. Cell concentration was determined using the Countess II FL Automated Cell Counter (Thermo Fisher Scientific). Trypan blue staining of the methanol fixed cells showed that 100% of the cells were dead, indicating that all cells were effectively fixed and permeabilized.

Chromium controller and Chromium NextGEM Single Cell 3’ Reagents Kit v3.1 (10× Genomics) have been used for partitioning cells into Gel Beads-in-Emulsion, in which all generated cDNA share a common 10× barcode. Libraries were generated from the cDNA following manufacturer’s instruction and checked with both Qubit 2.0 Fluorometer (Invitrogen) and Agilent Bioanalyzer DNA assay (Agilent Technologies). Libraries were then prepared for sequencing and sequenced on NovaSeq6000 (Illumina) with the following run parameters: Read 1 = 28 cycles, i7 index = 8 cycles, and Read 2 = 91 cycles.

### scRNA-Seq data processing.

The raw sequencing data were processed by Cell Ranger v 3.1.0 (10× Genomics) with mouse transcriptome reference mm10 to generate gene cell expression matrices. Further data analysis was carried out in R version 3.6.0 using Seurat version 3.1.1 (46). The 2 data sets were set up as independent Seurat objects. ‘‘Cells’’ that fit any of the following criteria were filtered out: <200 or >4500 expressed genes or >10% UMIs mapped to mitochondria. Data set normalization and identification of variable features were performed using the NormalizeData() function and the FindVariableFeatures() with following parameters (selection.method = “vst”, nfeatures = 4000). Integration anchors were computed using the first 20 dimensions, using all the genes present in both data sets as features to integrate. Finally, we obtained 3949 cells that passed quality control, with an average of 1460 genes expressed per cell. For downstream integrated analyses, top 30 components were used for PCA, UMAP, and cluster identification (using a resolution of 0.4). Further, we manually assigned cell population identity based on cell type–markers and merged those clusters that displayed similar canonical markers. After clustering and cell population identification, the most highly differentially expressed genes, or putative cluster markers, were identified by a likelihood-ratio test using the FindAllMarkers() function with the following parameters (only.pos = TRUE, min.pct = 0.5, min.diff.pct = 0.25, logfc.threshold = 0.25). Genes differentially expressed in CTR versus DEN where identified using the FindMarkers() function and subsequently filtered using the following criteria: (pct.1>0.45 or pct.2>0.45; p_val_adj < 0.01; avg_logFC < (–0.58) or avg_logFC>0.58). Data set integration with previously published scRNA-Seq (Giordani and De Micheli, refs. 22–24) was performed in Seurat with FindIntegrationAnchors() and IntegrateData() functions using the first 20 dimensions. From the De Micheli data set (24), only the uninjured data point (“D0”) was used for comparison. Both data sets were downloaded from the GEO website.

### Figure design.

Graphical abstract, Figure 1D, Figure 4A, and Figure 6, B and E, were created with BioRender (https://biorender.com/).

### Data and code availability.

a7int^+^ mouse bulk RNA-Seq data, mouse scRNA-Seq data, and Ngfr^+^ mouse bulk RNA-Seq data are available at the SRA repository (accession PRJNA623246, PRJNA626530, and PRJNA649152).

### Statistics.

Data are presented as mean ± SD. Statistical analysis was performed using Graph Pad Prism 8.0 software. Normality was assessed by Shapiro-Wilk test. Unpaired, 2-tailed Student’s *t* test was used to compare the means of 2 parametric groups, whereas Mann-Whitney test for 2 nonparametric groups. One-way ANOVA with Tukey’s post test was used for comparison among the different parametric data sets. *P* values of less than 0.05 were considered significant.

The number of biological replicates for each experiment is indicated in the figure legends. RNA-Seq data was performed in 2 independent samples derived from different animals. Statistical method was Deseq2. Right-tailed Fisher’s exact test and 1-sided Fisher’s exact test was used for IPA analyses. For scRNA-Seq, biological sample replicates were obtained from separate mice. Histological and Immunofluorescence images are representative of at least 3 different experiments/animals. For cell culture studies, biological replicates were from separate culture wells.

### Study approval.

All experiments in this study were performed in accordance with protocols approved by the Italian Ministry of Health, Rome, Italy; Santa Lucia Foundation (Rome, Italy); and the Sanford Burnham Prebys Medical Discovery IACUC. The study is compliant with all relevant ethical regulations regarding animal research and in the respect to the principles of the 3Rs (Replacement, Reduction, and Refinement).

## Author contributions

DP, PLP, and LM designed the research studies. DP and LM conducted the experiments. DP, LM, MD, LG, CD, and SM conducted the investigation. LM, AS, MA, MB, CV, and PLP provided the resources. DP, LG, SA, GB, and LM analyzed the data. LM created the original draft of the manuscript. LM, LG, PLP, BLO, CV, and MB reviewed and edited the manuscript. LM and PLP supervised the project. LM and PLP managed the project administration and funding acquisition.

## Supplementary Material

Supplemental data

## Version 1 03/04/2021

In-Press Preview

## Version 2 04/08/2021

Electronic publication
