# Supplementary material for: Activation of skeletal muscle–resident glial cells upon nerve injury
Source: JCI Insight. 2021 Apr 8;6(7):e143469. doi: 10.1172/jci.insight.143469 (PMC8119188; doi:10.1172/jci.insight.143469)
Supplement: Supplemental data [file jciinsight-6-143469-s163.pdf]

SUPPLEMENTAL DATA

Activation of skeletal muscle-resident glial cells upon nerve injury

Proietti D.[1-2], Giordani L.[3], De Bardi M.[1], Chiara D'Ercole[1], Lozanoska-Ochser B.[2], Amadio S.[1], Volontè C.[1-4], Marinelli S.[5], Muchir A.[6], Bouchè M.[2], Borsellino G.[1], Sacco A.[7], Puri PL.[7] and Madaro L.[2-8]*

Supplementary Figure 1

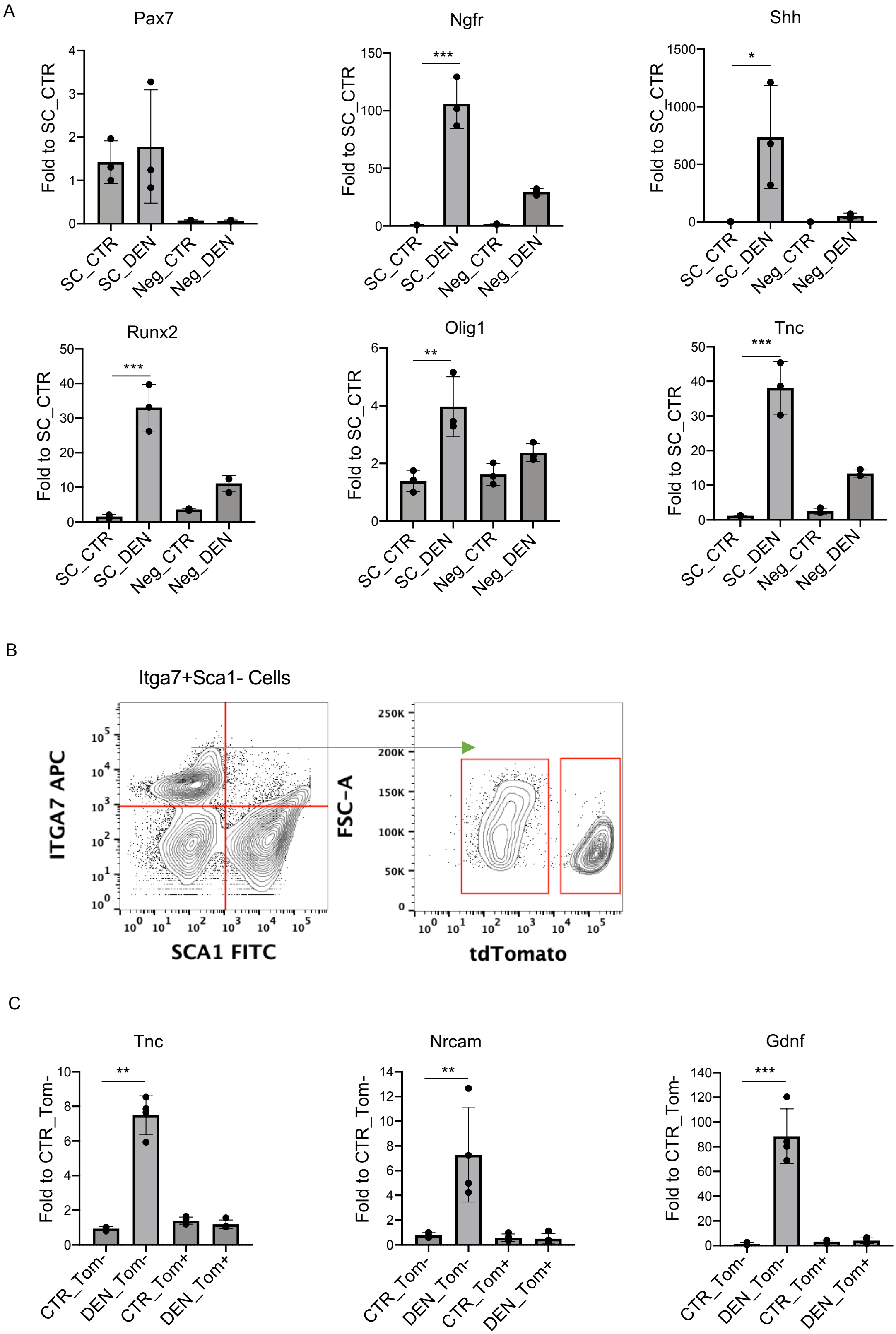

Supplementary Figure 1.

A) qPCR analysis for the expression of Pax7, Ngfr, Shh, Runx2, Olig1 and Tnc in freshly isolated cells from control and denervated muscle isolated with *Satellite Cells isolation kit – Miltenyi Biotec* (SC) or the negative fraction (Neg). Gapdh was used as housekeeping gene (n=3, Values represent mean ± s.d. *P < 0.05, **P < 0.01 and ***P <0.001; by One Way Anova Tukey's Multiple Comparisons test). **B)** FACS plot profile of Itga7+ cells freshly isolated from tamoxifen treated PAX7.Cre_tdTomato mice. **C)** qPCR analysis for the expression of Tnc, Nrcam and Gdnf in freshly isolated Tomato+ and Tomato- cells derived from control and 3 days denervated muscle of tamoxifen treated PAX7.Cre_tdTomato mice. Gapdh was used as housekeeping gene (n=4, Values represent mean ± s.d. **P < 0.01 and ***P <0.001; by One Way Anova Tukey's Multiple Comparisons test).

Supplementary Figure 2

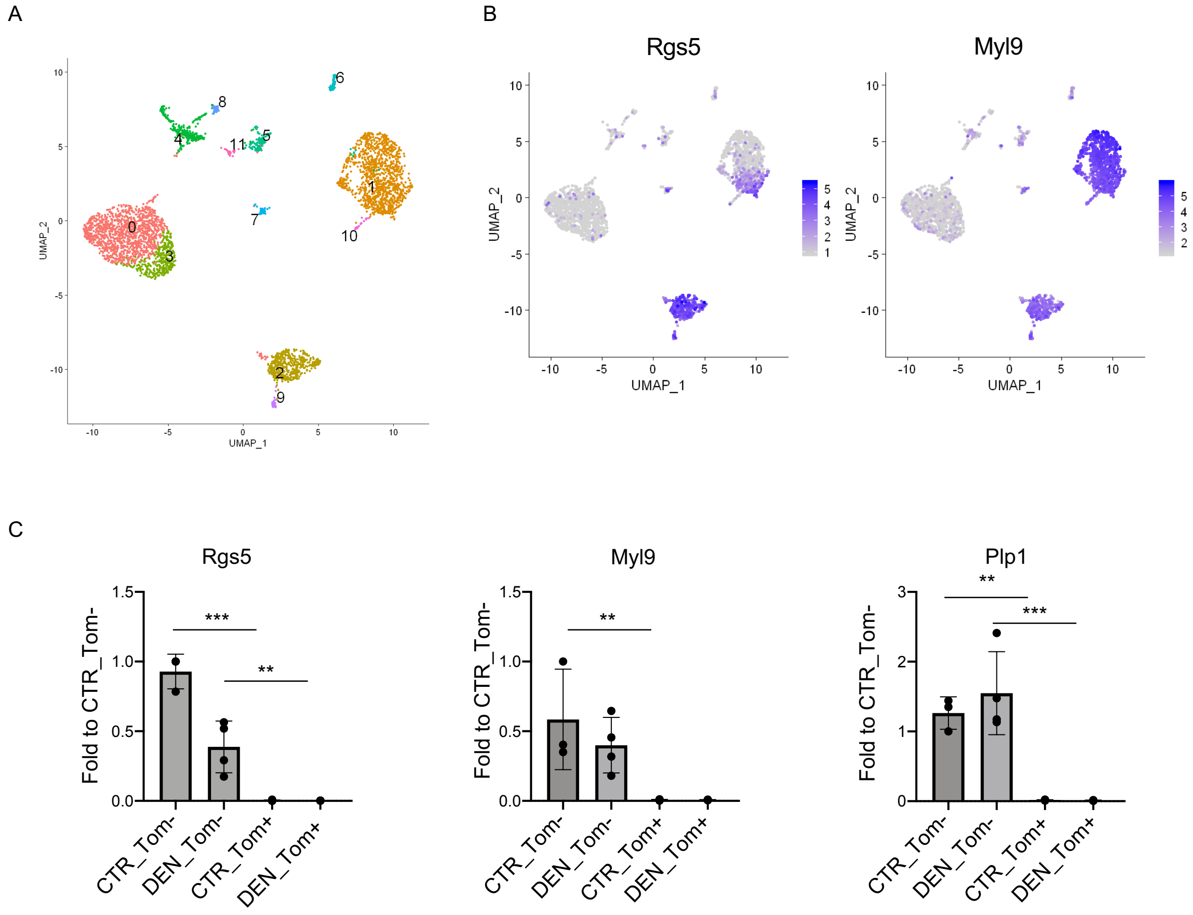

Supplementary Figure 2.

A) Uniform Manifold Approximation and Projection (UMAP)-derived clusters of single cells (Single Cells RNA-seq) of Itga7+Sca1-Ln- isolated cells from control muscle. **B)** Distribution of Rgs5 and Myl9 transcripts in Uniform Manifold Approximation and Projection (UMAP)-derived clusters of single cells (Single Cells RNA-seq) of Itga7+Sca1-Ln- isolated cells from control muscle. **C)** qPCR analysis for the expression of Rgs5, Myl9 and Plp1 in freshly isolated Tomato+ and Tomato- cells derived from control and 3 days denervated muscle of tamoxifen treated PAX7.Cre_tdTomato mice. Gapdh was used as housekeeping gene (n=4, Values represent mean ± s.d. $^{**}P < 0.01$ and $^{***}P < 0.001$; by One Way Anova Tukey's Multiple Comparisons test).

Supplementary Figure 3

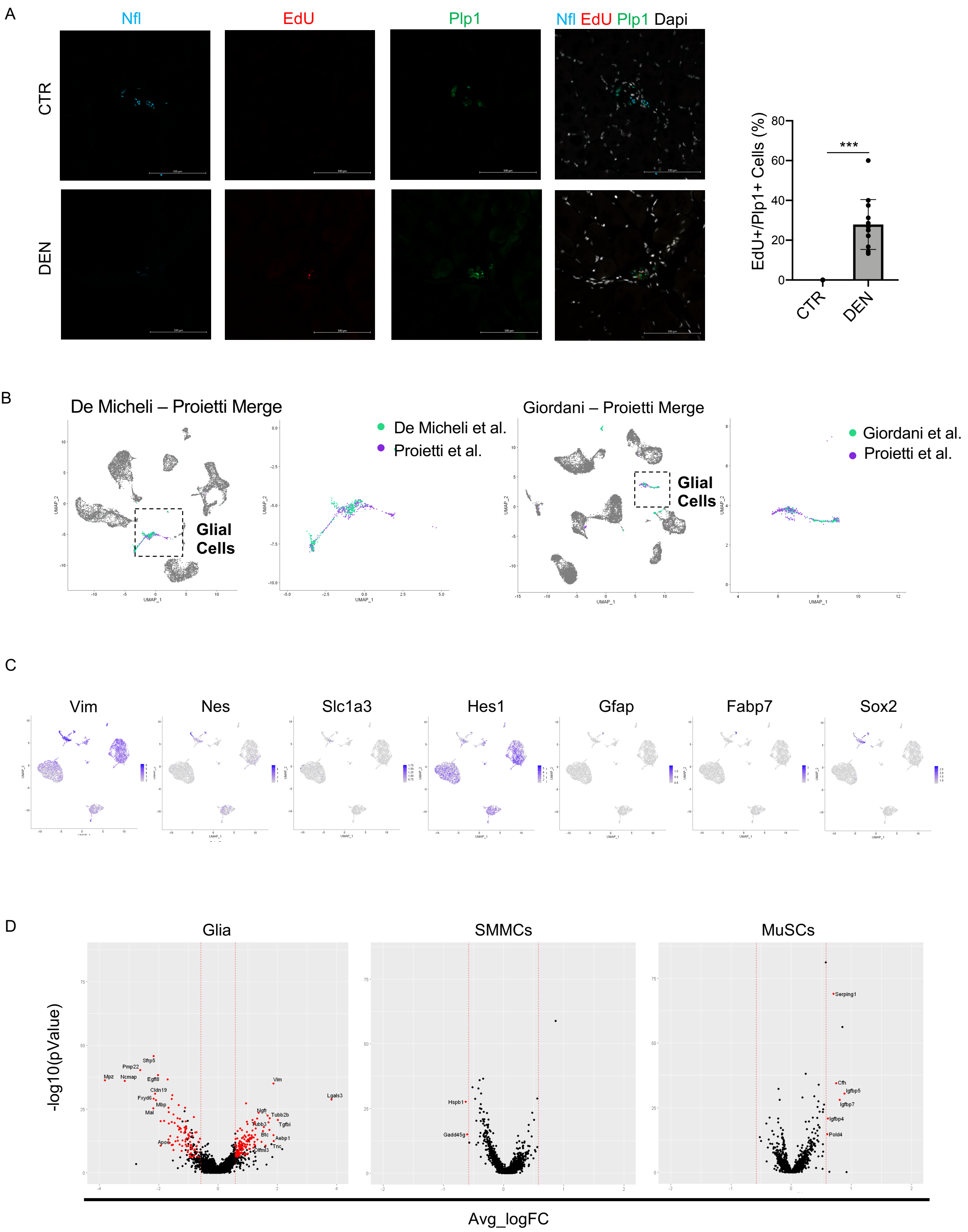

Supplementary Figure 3.

A) Representative immunofluorescence analysis of TA muscle cryosection derived from control and denervated muscles, stained for Neurofilament-L (Nfl, Cyan), EdU (red) and Plp1 (green). Nuclei were counterstained with dapi. Scale bar = 100μm. (right) Graph representing the percentage of Plp1+EdU+ cells in cryosection of control and denervated muscle. (n=9 slide of 3 different animals. ***P < 0.001; by student t-test) **B)** Uniform Manifold Approximation and Projection (UMAP)-derived clusters of single cells. Single Cells RNA-seq overlap of Plp1 cluster with previously published dataset from uninjured muscle (De Micheli et al. – Day 0 and Giordani et al. **C)** Distribution of Vim, Nes, Slc1a3, Hes1, Gfap, Fabp7 and Sox2 transcripts in Uniform Manifold Approximation and Projection (UMAP)-derived clusters of single cells (Single Cells RNA-seq) of Itga7+Sca1-Ln- isolate cells from control muscle. **D)** Volcano plots for differentially expressed transcripts represents the logarithm of average fold change (Avg_logFC) on x-axis and log10 of the q-value of each transcripts on y-axis; The significative changes induced by denervation was shown for Glia, SMMCs and MuSCs.

Supplementary Figure 4

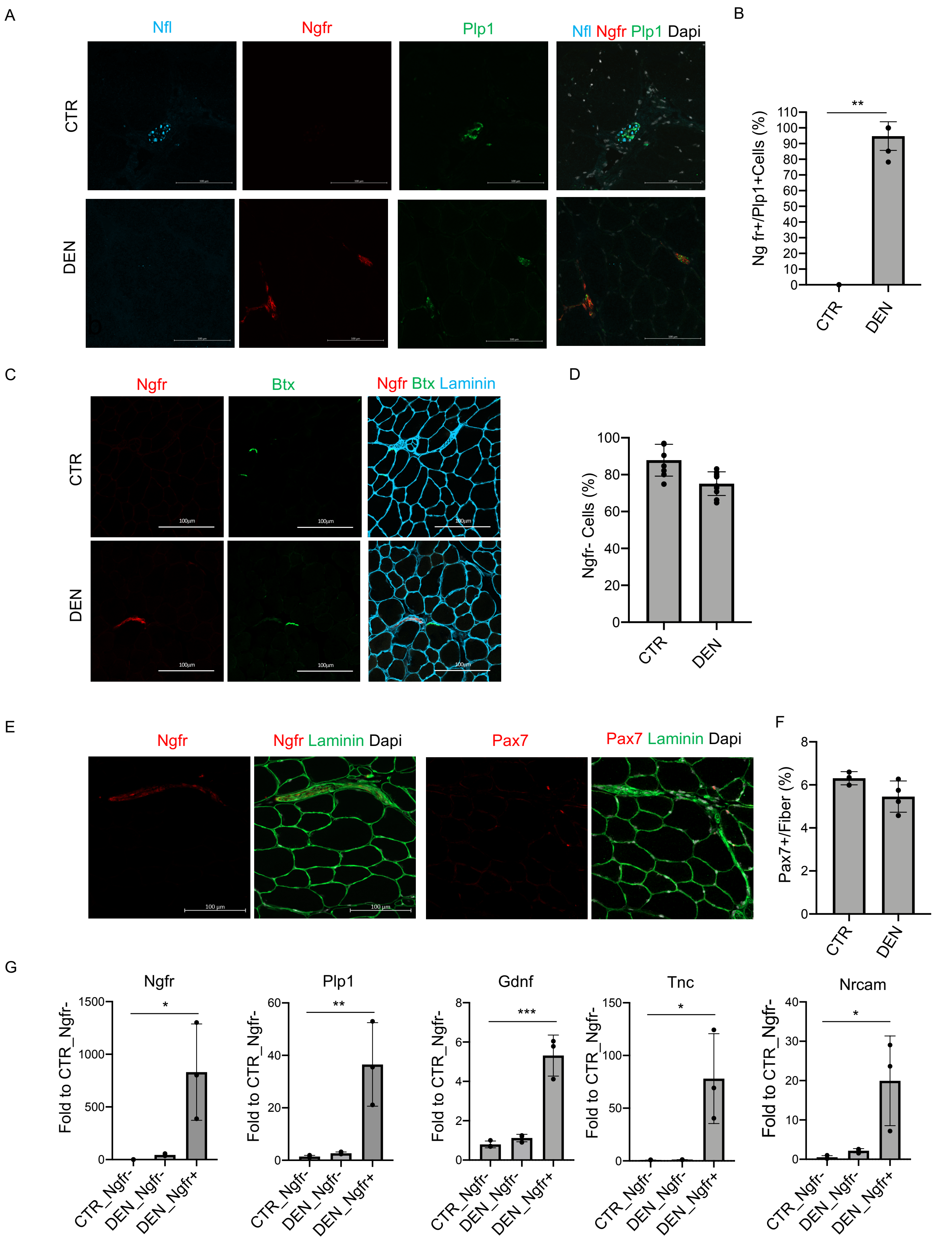

Supplementary Figure 4.

A) Representative immunofluorescence analysis of TA muscle cryosection derived from control (CTR) and denervated (DEN) muscle, stained for Neurofilament-L (Nfl, Cyan), Ngfr (red), Plp1 (green). Nuclei were counterstained with dapi. Scale bar = 100μm. **B**) Quantification of Ngfr+/Plp1+ cells in control (CTR) and denervated (DEN) muscle (n=7, Values represent mean ± s.d. **P < 0.01; by Mann-Whitney test) **C)** Representative immunofluorescence analysis of TA muscle cryosection derived from control (CTR) and denervated (DEN) muscle, stained for Ngfr (Red), Bungarotoxin (Green) and Caveolin-3 (Cyan). Nuclei were counterstained with dapi. Scale bar = 100μm. **D)** Quantification of Itga7+Ngfr- cells was shown in the graphs (n=8 CTR, n=10 DEN, Values represent mean ± s.d.). **E)** Representative immunofluorescence analysis of TA muscle cryosection derived from denervated muscle, stained for Ngfr (red, left), Pax7 (red, right), Laminin (green). Nuclei were counterstained with dapi. Scale bar = 100μm. **F)** Quantification of Pax7+ cells in control and denervated muscles (n=3, Values represent mean ± s.d.). **G)** qPCR analysis for the expression of Ngfr, Plp1, Gdnf, Tnc, Nrcam in freshly isolated Ngfr+ or Ngfr- cells derived from control and 3 days denervated muscle. Gapdh was used as housekeeping gene (n=3, Values represent mean ± s.d. *P < 0.05, **P < 0.01 and ***P <0.001; by One Way Anova Tukey’s Multiple Comparisons test).

Supplementary Figure 5

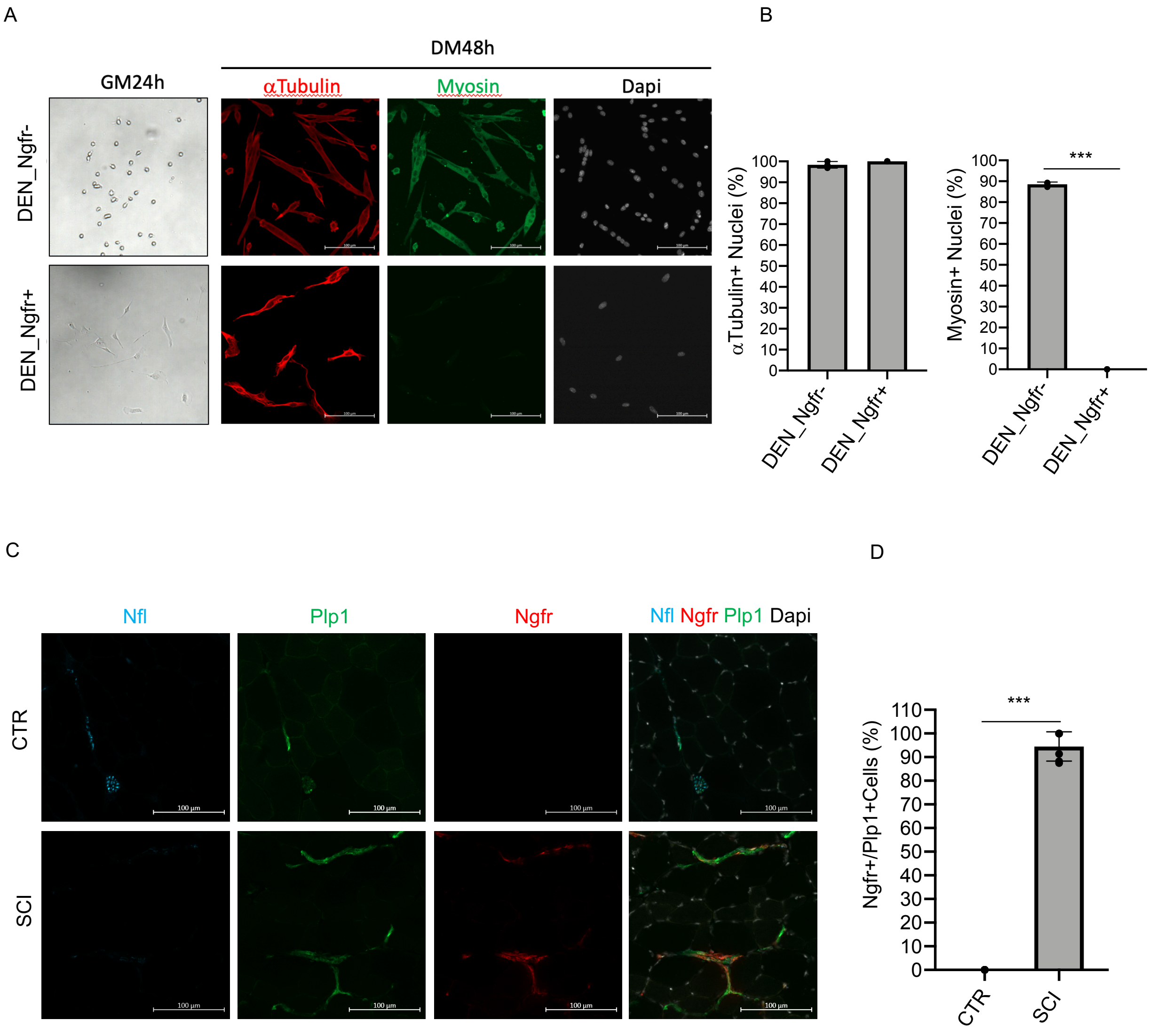

Supplementary Figure 5.

A) *In-vitro* culture of both Ngfr- and Ngfr+ cells from denervated muscle. Phase contrast images of proliferating cells was shown (left panel). Representative immunofluorescence analysis for αTubulin (red) and Myosin (green) was performed 48h after myogenic differentiation. Nuclei were counterstained with dapi. Scale bar = 100μm. **B)** Quantification of αTubulin+ (left) and Myosin+ (right) nuclei (n=3, Values represent mean ± s.d. ***P < 0.001; by student t-test). **C)** Representative immunofluorescence analysis of control and spinal cord injury (SCI) mice muscle cryosections, at 7-days post injury, stained for Neurofilament-L (Nfl, Cyan), Ngfr (red) and Plp1 (green). Nuclei were counterstained with dapi. Scale bar = 100μm. **D)** Quantification of Ngfr+/Plp1+ cells in control (CTR) and spinal cord injury (SCI) mice muscle (n=6, Values represent mean ± s.d. ***P < 0.001; by Mann-Whitney test).

Supplementary Figure 6

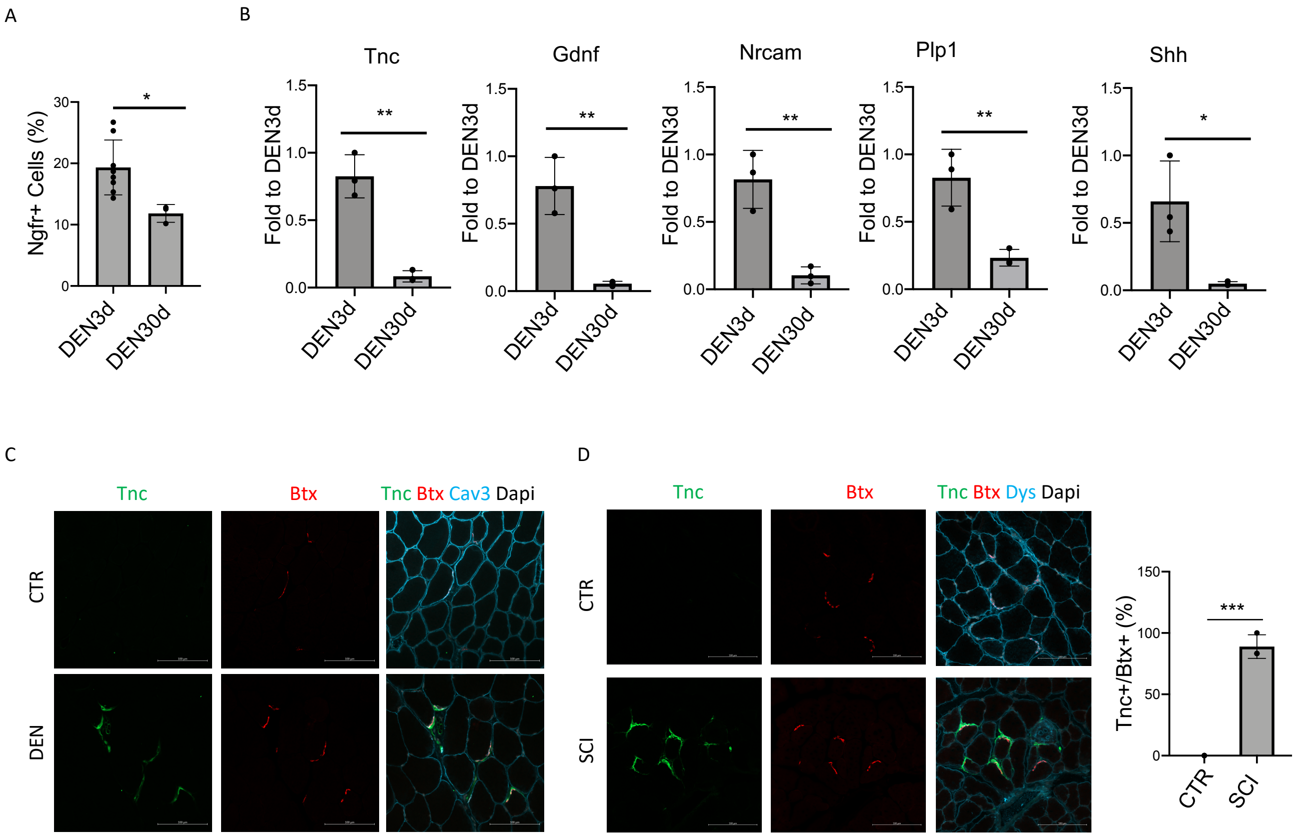

Supplementary Figure 6.

A) Ngfr+ cell quantification, shown in the graphs as a percentage of Itga7+Sca1-Ln- population, in denervated muscle at 3- and 30-days post nerve lesion ($n \geq 3$, Values represent mean ± s.d. *$P < 0.05$; by student t-test). **B)** qPCR analysis for the expression of Tnc, Gdnf, Nrcam, Plp1 and Shh in freshly isolated Ngfr+ cells derived from denervated muscle at 3- and 30-days post nerve lesion (n=3, Values represent mean ± s.d. *$P < 0.05$ and **$P < 0.01$; by student t-test). **C)** Representative immunofluorescence analysis of TA muscle cryosection derived from control and denervated muscle, stained for Tnc (green), Bungarotoxin (Btx, red) and Caveolin-3 (Cav3, Cyan). Nuclei were counterstained with dapi. Scale bar = 100μm. **D)** Representative immunofluorescence analysis of control and spinal cord injury (SCI) mice muscle cryosection, at 7-days post injury, stained for Tnc (green), Bungarotoxin (Btx, red) and Dystrophin (Dys, Cyan). Nuclei were counterstained with dapi. Scale bar = 100μm. Quantification of the co-localization Bungarotoxin (Btx) and Tnc data on the right (n=3, Values represent mean ± s.d. ***$P < 0.001$; by student t-test).

Supplementary Figure 7

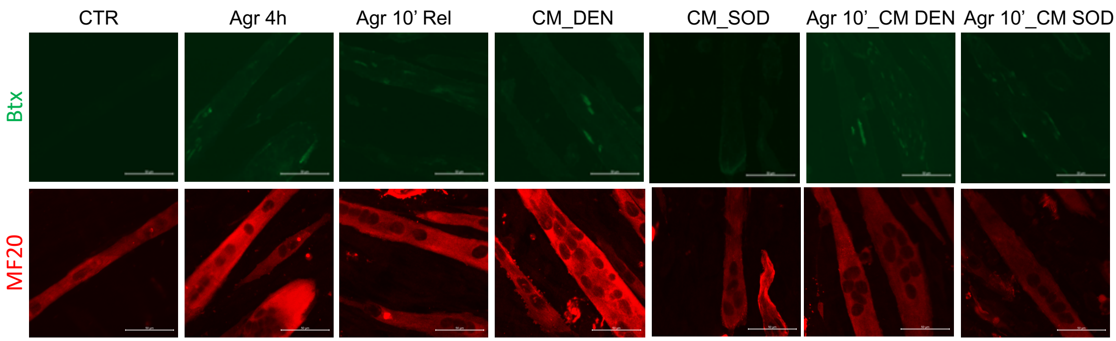

Supplementary Figure 7.

a) Representative immunofluorescence analysis of C2C12 myotubes stained for Bungarotoxin (Btx, green) and Myosin (MF20, red). Scale bar = 50μm.
